# Pulmonary symptoms and diagnoses are associated with HIV in the MACS and WIHS cohorts

**DOI:** 10.1186/1471-2466-14-75

**Published:** 2014-04-30

**Authors:** Matthew R Gingo, Goundappa K Balasubramani, Thomas B Rice, Lawrence Kingsley, Eric C Kleerup, Roger Detels, Eric C Seaberg, Ruth M Greenblatt, Susan Holman, Laurence Huang, Sarah H Sutton, Marnie Bertolet, Alison Morris

**Affiliations:** 1 Department of Medicine, School of Medicine, University of Pittsburgh, Pittsburgh, PA, USA; 2 Department of Epidemiology, Epidemiology Data Center, Graduate School of Public Health, University of Pittsburgh, Pittsburgh, PA, USA; 3 Department of Infectious Diseases and Microbiology, Graduate School of Public Health, University of Pittsburgh, Pittsburgh, PA, USA; 4 Department of Medicine, David Geffen School of Medicine, University of California, Los Angeles, CA, USA; 5 Department of Epidemiology, Bloomberg School of Public Health, Johns Hopkins University, Baltimore, MD, USA; 6 Department of Clinical Pharmacy, School of Pharmacy, University of California, San Francisco, CA, USA; 7 Department of Medicine, School of Medicine, University of California, San Francisco, CA, USA; 8 Department of Epidemiology and Biostatistics, School of Medicine, University of California, San Francisco, CA, USA; 9 Department of Medicine, SUNY Downstate Medical Center, Brooklyn, NY, USA; 10 Department of Medicine, Feinberg School of Medicine, Northwestern University, Chicago, IL, USA; 11 Department of Immunology, School of Medicine, University of Pittsburgh, Pittsburgh, PA, USA

**Keywords:** AIDS, HIV, Pulmonary disease, Chronic obstructive, Respiratory tract diseases, Sleep apnea syndromes

## Abstract

**Background:**

Several lung diseases are increasingly recognized as comorbidities with HIV; however, few data exist related to the spectrum of respiratory symptoms, diagnostic testing, and diagnoses in the current HIV era. The objective of the study is to determine the impact of HIV on prevalence and incidence of respiratory disease in the current era of effective antiretroviral treatment.

**Methods:**

A pulmonary-specific questionnaire was administered yearly for three years to participants in the Multicenter AIDS Cohort Study (MACS) and Women’s Interagency HIV Study (WIHS). Adjusted prevalence ratios for respiratory symptoms, testing, or diagnoses and adjusted incidence rate ratios for diagnoses in HIV-infected compared to HIV-uninfected participants were determined. Risk factors for outcomes in HIV-infected individuals were modeled.

**Results:**

Baseline pulmonary questionnaires were completed by 907 HIV-infected and 989 HIV-uninfected participants in the MACS cohort and by 1405 HIV-infected and 571 HIV-uninfected participants in the WIHS cohort. In MACS, dyspnea, cough, wheezing, sleep apnea, and incident chronic obstructive pulmonary disease (COPD) were more common in HIV-infected participants. In WIHS, wheezing and sleep apnea were more common in HIV-infected participants. Smoking (MACS and WIHS) and greater body mass index (WIHS) were associated with more respiratory symptoms and diagnoses. While sputum studies, bronchoscopies, and chest computed tomography scans were more likely to be performed in HIV-infected participants, pulmonary function tests were no more common in HIV-infected individuals. Respiratory symptoms in HIV-infected individuals were associated with history of pneumonia, cardiovascular disease, or use of HAART. A diagnosis of asthma or COPD was associated with previous pneumonia.

**Conclusions:**

In these two cohorts, HIV is an independent risk factor for several respiratory symptoms and pulmonary diseases including COPD and sleep apnea. Despite a higher prevalence of chronic respiratory symptoms, testing for non-infectious respiratory diseases may be underutilized in the HIV-infected population.

## Background

With widespread use of highly active antiretroviral therapy (HAART) for treatment of HIV, the incidence of infectious complications of HIV and related mortality has declined [1,2]. Although treatment has led to increased longevity in HIV-infected persons, there may be an increase in morbidity and mortality secondary to non-AIDS related conditions [3-5]. Chronic lung conditions may contribute to morbidity and mortality as conditions such as chronic obstructive pulmonary disease (COPD), [1,6,7] bronchogenic carcinoma, [8] pulmonary hypertension, [9] and pulmonary fibrosis [1] have been reported to be more prevalent in HIV-infected persons. Prior studies have been based largely on medical record review, and little is known about the prevalence of respiratory symptoms, degree of diagnostic testing related to cardiopulmonary disease, and prevalence and incidence of patient-reported diagnoses related to chronic lung disease in HIV-infected persons during the current HAART era.

The increased prevalence of risk behaviors such as cigarette smoking and illicit drug use in the HIV population [10-12] make it difficult to determine the independent effect of HIV infection on occurrence of lung disease. Prior to HAART, studies showed that HIV was associated with a greater prevalence of respiratory symptoms and emphysema [13,14]. Recent studies have shown respiratory symptoms and pulmonary disease to be common in HIV-infected persons, [1,15-19] but these studies are limited as they are based on administrative data, do not address symptoms or performance of diagnostic testing, or lack an appropriate HIV-uninfected group for comparison.

In order to assess the magnitude of chronic respiratory disease and symptoms related to HIV infection, we surveyed the participants of the Multicenter AIDS Cohort Study (MACS) and the Women’s Interagency HIV Study (WIHS), two large cohorts of HIV-infected and HIV-uninfected individuals. We tested the hypothesis that respiratory symptoms and chronic lung diseases are more common in HIV-infected persons and identified factors related to a diagnosis of lung disease in HIV-infected individuals.

## Methods

### Participants

The MACS and WIHS cohorts have been described previously [20,21]. Briefly, the MACS is a prospectively followed cohort of 6,972 men with or at risk for HIV started in 1984, and the WIHS is a prospectively followed cohort of 3,766 women with or at risk for HIV started in 1994. The MACS is primarily a cohort of men who have sex with men, and WIHS is a cohort of women who engage in high risk behavior for HIV infection, primarily intravenous drug use. Participants in this study were HIV-infected and HIV-uninfected individuals who completed a pulmonary questionnaire at an ongoing MACS or WIHS study visit between January 2008 and December 2010. All participants were eligible. If participants did not complete a baseline pulmonary questionnaire during the 2008 visit cycle, they were excluded from further study. All participants gave written informed consent to study protocols approved by the institutional review boards of participating sites (University of Pittsburgh; Women’s Interagency HIV Study Data Management and Analysis Center; Johns Hopkins University; University of California, Los Angeles; University of California, San Francisco; State University of New York Downstate Medical Center; Northwestern University; and the Center for Analysis and Management of Multicenter AIDS Cohort Study).

### Data collection

At yearly MACS or WIHS visits from 2008 to 2010, participants responded to a modified American Thoracic Society-Division of Lung Disease-78 Respiratory Disease Questionnaire [22] that collected information regarding frequency of respiratory symptoms (cough, phlegm production, wheezing, and dyspnea) and prevalent (2008) and then incident (2009, 2010) therapies (medication, oxygen therapy, nocturnal non-invasive ventilation), diagnostic testing (self-report of having had sputum sampling, bronchoscopy, chest computed tomography [CT], pulmonary function testing, echocardiography, or polysomnography), and chronic diagnoses (self-report of having been told they had asthma, COPD, chronic bronchitis, lung fibrosis, sarcoidosis, pulmonary hypertension, pulmonary emboli, and sleep apnea). Baseline pulmonary questionnaire asked if the participant had ever had diagnostic testing, and follow-up pulmonary questionnaires if they had diagnostic testing in the past year. Participants were considered to have significant respiratory symptoms if they reported that symptoms occurred more frequently than just with colds or infections and were experienced at least monthly. The presence of any COPD was defined as having been told they had a diagnosis of COPD, chronic bronchitis, or emphysema.

Time-dependent data were determined from the visit at which the prevalence pulmonary questionnaire was administered (2008) and included age, HIV status, duration of HIV infection, body mass index (BMI), cigarette smoking status, cumulative pack-years of cigarette smoking, alcohol consumption, use of intravenous drugs in the past 6 months, use of cocaine in the past 6 months, diagnosis of cardiac disease (by self-report of myocardial infarction, hospitalization for congestive heart failure hospitalization for chest pain/angina, surgery on heart vessels, or other heart disease) use of HAART, current CD4 count, and current plasma HIV RNA level. Data from all visits prior to and including baseline visit were used to determine participants’ ever-use of intravenous drugs or cocaine, history of tuberculosis, history of *Pneumocystis* or bacterial pneumonia, duration of HAART use, and mean and nadir CD4 counts. HIV status was determined by HIV serological testing at each visit in previously seronegative participants, and participants who were HIV positive at baseline visit were assumed to have duration of HIV infection from time of MACS or WIHS enrollment to the date corresponding to the prevalence pulmonary questionnaire visit. Race, ethnicity, cigarette smoking history, alcohol consumption, and illicit drug use were determined by participant self-report. Alcohol consumption was categorized as none, light drinking (<3 drinks/week), moderate drinking (3-13 drinks/week), or heavy drinking (≥14 drinks/week). HAART was defined previously [23] and categorized as never, past HAART use, or current HAART use.

### Statistical analysis

The MACS and WIHS cohorts were analyzed separately because of variations in data collection procedures and differences in the populations sampled, including gender, proportions of minorities, socioeconomic status, access to health care, and substance use. To assess selection bias, characteristics (age, race/ethnicity, smoking history, alcohol use, drug use, HIV status, and CD4 counts and viral loads) were compared between participants in each cohort who completed the prevalence pulmonary questionnaire to those who were enrolled in the cohort and did not complete the questionnaire. For participants who had not completed the prevalence pulmonary questionnaire because they failed to show for the scheduled visit, data were carried forward from the last visit they had attended.

Personal characteristics, clinical data, presence of respiratory symptoms, medical therapies, diagnostic testing, and diagnoses were compared between HIV-infected and HIV-uninfected groups in each cohort using parametric (t-tests) and non-parametric (chi-square, Wilcoxon) tests as appropriate. Respiratory symptoms, medical therapies, diagnostic testing, and diagnoses were also compared between HIV-infected and HIV-uninfected groups in each cohort stratified by smoking status because cigarette smoking is such a dominant risk factor for lung disease. To determine incidence of pulmonary diagnoses from follow-up questionnaires (2009-2010), incidence rates for specific pulmonary diseases were computed in those who did not report that disease at the prevalence pulmonary questionnaire (2008) as the number of observed incident events divided by the number of person-years of follow-up, where the follow-up time available for each person was the number of years from the initial pulmonary evaluation visit until the earliest of the following: disease diagnosis date, death, loss to follow-up, or the date of the last visit with a pulmonary questionnaire.

Multivariable modeling was then performed for the most prevalent conditions: symptoms, diagnostic testing, asthma, COPD, and sleep apnea. Poisson regression methods [24] were used to examine the association of baseline measures and HIV status with baseline prevalence of respiratory symptoms, diagnostic testing, and diagnoses, and to identify potential confounders. Baseline characteristics examined included age, African-American race, Hispanic ethnicity, smoking status, pack-years smoked, alcohol status, intravenous drug use ever, cocaine use ever, and hepatitis C status. Adjusted prevalence ratio models were fitted with robust variance to quantify the association of HIV status with the outcomes independent of the effect of race, smoking status, intravenous drug use ever, age, and any additional significant factors (p < 0.05) added from the above multivariable model. Similar models were repeated restricting to HIV-infected participants to assess HAART use, CD4 cell counts, and plasma HIV RNA levels, and an additional set of models restricted to HIV-infected participants were performed to include history of cardiovascular disease and history of *Pneumocystis* or bacterial pneumonia to assess for contributions of these variables. We also assessed if protease inhibitors were related to symptoms or diagnoses by including protease inhibitor use in the model instead of HAART use, but there were no associations with this specific antiretroviral category (data not shown). To evaluate the associations between incident diagnoses during the two-year follow-up and HIV status, we fit separate multivariable Poisson regression models to incidence data [25,26]. For covariates with similar origin (for example current or ever using intravenous drugs or the current or mean CD4 count), the variable with the lowest bivariate p-value was included in the model. Prevalence ratios or rate ratios and 95% confidence intervals were reported for all outcomes. An interaction between HIV infection and smoking status was also test by adding an interaction term to the final multivariable models. A p-value <0.05 determined statistical significance. We analyzed data using SAS Version 9.2 (SAS, Inc., Cary, NC).

## Results

### Participants

The prevalence pulmonary questionnaire was completed at a study visit in 2008 by 907 HIV-infected and 989 HIV-uninfected participants in the MACS cohort and 1405 HIV-infected and 571 HIV-uninfected participants in the WIHS cohort. There were 223 MACS participants and 488 WIHS participants who did not complete the prevalence pulmonary questionnaire (Additional file 1: Table S1). In both cohorts, equal proportions of HIV-infected participants completed the questionnaire. Participants who completed the questionnaire were more likely to be older, Caucasian, use recreational drugs, and have lower plasma HIV RNA levels. In WIHS, there were fewer never-smokers and more never drinkers of alcohol in participants who completed the questionnaire, and Hispanics were more likely to complete the questionnaire.

There were significant differences in demographic and clinical factors between the HIV-infected and HIV-uninfected participants in each cohort at the time of the prevalent questionnaire (Table 1). In the MACS cohort, HIV-infected individuals were younger, more likely to be African-American, smoked more, and used cocaine more than HIV-uninfected individuals; while in the WIHS cohort, HIV-infected individuals were older, less likely to be African-American, and less likely to smoke than HIV-uninfected participants. HIV-infected participants in both cohorts had lower BMI and were less likely to drink, but were more likely to use intravenous recreational drugs, be hepatitis C seropositive, and have had pneumonia compared to HIV-uninfected individuals.

**Table 1:** Participant characteristics

	**MACS**			**WIHS**		
	**HIV-uninfected**	**HIV-infected**		**HIV-uninfected**	**HIV-infected**	
	**N = 989**	**N = 907**	**p-value**	**N = 571**	**N = 1405**	**p-value**
Age, mean (SD)	52.3 (10.7)	49.2 (8.8)	<0.001	40.8 (10.1)	44.4 (8.7)	<0.001
African American, n (%)	185 (18.7)	297 (32.8)	<0.001	363 (63.6)	820 (58.4)	0.03
Hispanic, n (%)	72 (7.3)	98 (10.8)	0.007	149 (26.1)	392 (27.9)	0.41
BMI, n (%)	27.2 (5.1)	25.6 (4.4)	<0.001	30.6 (8.4)	28.1 (7.4)	<0.001
Smoking status, n (%)			<0.001			<0.001
Never	258 (26.3)	222 (24.8)		136 (23.9)	441 (31.8)	
Former	499 (50.9)	395 (44.1)		139 (24.4)	404 (29.2)	
Current	223 (22.8)	278 (31.1)		295 (51.7)	540 (39.0)	
Pack years, median (Q1-Q3)	0.5 (0-20)	4 (0-21)	0.02	13 (0.5-25.5)	13.2 (0-26.1)	0.28
Alcohol use, n (%)			<0.001			<0.001
None	155 (15.8)	201 (22.5)		274 (48.1)	896 (64.7)	
Light	463 (47.3)	477 (53.4)		178 (31.2)	351 (25.3)	
Moderate	264 (27.0)	165 (18.5)		93 (16.3)	91 (6.6)	
Heavy	97 (9.9)	50 (5.6)		25 (4.4)	47 (3.4)	
Intravenous drug use-ever, n (%)	88 (8.9)	145 (16.0)	<0.001	119 (20.8)	376 (26.8)	0.006
Cocaine use-ever, n (%)	427 (43.2)	520 (57.3)	<0.001	329 (57.6)	773 (55.0)	0.29
Hepatitis C positive, n (%)	73 (7.4)	138 (15.2)	<0.001	105 (18.5)	405 (28.9)	<0.001
Cardiovascular diagnosis ever, n (%)	67 (6.8)	80 (8.8)	0.1	29 (5.1)	47 (3.4)	0.07
Bacterial pneumonia ever, n (%)	43 (4.3)	109 (12.0)	<0.001	31 (5.4)	250 (17.8)	<0.001
*Pneumocystis* pneumonia ever, n (%)	-	49 (5.4)	-	1 (0.2)	150 (10.7)	<0.001
*M. tuberculosis* infection ever, n (%)	1 (0.1)	2 (0.2)	0.51	14 (2.5)	97 (6.9)	<0.001
Antiretroviral medication use, n (%)						
None	-	150 (16.8)	-	-	326 (23.2)	-
Prior HAART	-	43 (4.8)	-	-	8 (0.6)	-
Current HAART	-	702 (78.4)	-	-	1071 (76.2)	-
HAART duration, mean (SD)	-	14.1 (7.9)	-	-	15.5 (8.3)	-
Protease inhibitor use ever, n (%)	-	636 (70.1)	-	-	1024 (72.9)	-
CD4 counts (cells/uL), mean (SD)						
Current, at baseline pulmonary questionnaire	932.6 (311.5)	571.8 (277.4)	<0.001	1039.9 (331.3)	502 (291.2)	<0.001
Mean over the period followed in cohort	971.4 (274.6)	579.9 (230.7)	<0.001	1059.1 (309.6)	490.2 (217.8)	<0.001
Nadir during period followed in cohort	622.7 (224.9)	259.5 (168.6)	<0.001	728.8 (269.9)	209.9 (156.2)	<0.001
Plasma HIV RNA level (copies/mL), median (Q1-Q3)	-	40 (40-136)	-	-	80 (80-2400)	-
Plasma HIV RNA undetectable, n (%)*		590 (70.0)			764 (56.0)	

*HIV RNA level at baseline pulmonary questionnaire visit available in 843 MACS and 1365 WIHS participants. Undetectable level was <40 copies/mL in the MACS cohort and <80 copies/mL in the WIHS cohort.

BMI – body mass index; MACS - Multicenter AIDS Cohort Study; WIHS - Women’s Interagency HIV Study; SD - standard deviation; n - number; % - percentage; Q1 - quartile 1; Q3 - quartile 3.

The majority of HIV-infected participants in both cohorts were on HAART (78.4% and 76.2% in MACS and WIHS, respectively) at the time of the prevalence pulmonary questionnaire with a mean (SD) duration of HAART use of 14.1 (7.9) years in MACS and 15.5 (8.3) years in WIHS. HIV-infected participants had a mean (SD) CD4+ T-cell count at the time of the prevalence pulmonary questionnaire of 571.8 (277.4) cells/uL in MACS and 502 (291.2) cells/uL in WIHS, and the majority of participants had undetectable viral loads (MACS: plasma HIV RNA level < = 40copies/mL: 590/843 = 70%; WIHS: plasma HIV RNA level < =80 copies/mL: 764/1365 = 56%).

### Respiratory symptoms and treatments

**Figure 1:**
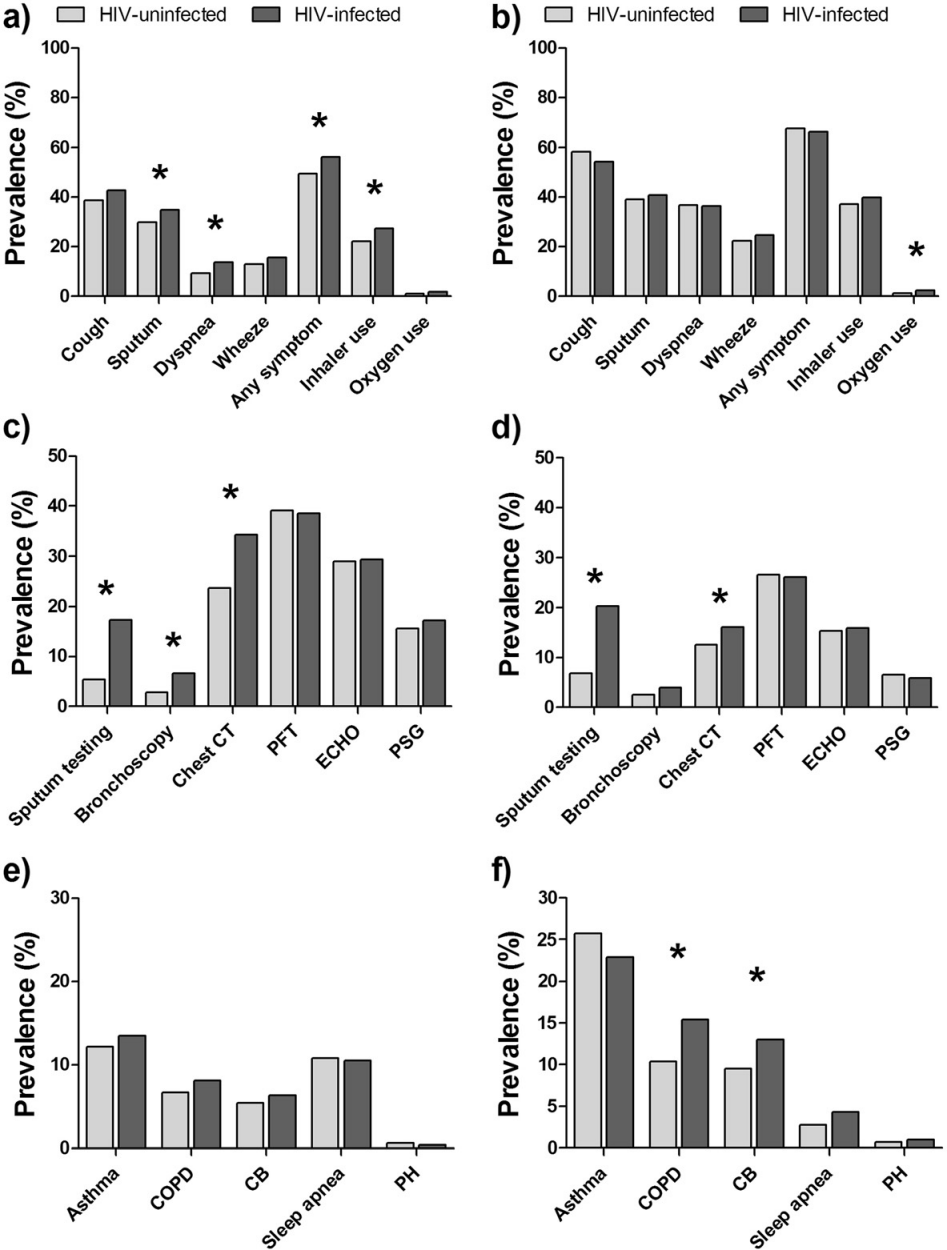
**Prevalence of respiratory symptoms in the Multicenter AIDS Cohort (MACS) (a) and Women’s Interagency HIV Study (WIHS) (b), diagnostic testing in MACS (c) and WIHS (d), and respiratory diagnoses in MACS (e) and WIHS (f) by HIV status.** ECHO - echocardiogram, CT - computed tomography, PFT - pulmonary function testing, PSG - polysomnography, COPD - chronic obstructive pulmonary disease, CB - chronic bronchitis, PH - pulmonary hypertension. (*) represents a p-value < 0.05 by chi-square test.

Respiratory symptoms and inhaler use were common in both cohorts at the time of the prevalence pulmonary questionnaire (Additional file 1: Table S2), and HIV was an independent risk factor for certain symptoms. In the MACS cohort, phlegm production, dyspnea, any respiratory symptoms, and inhaler use were reported more commonly by HIV-infected participants (Figure 1a), and in the WIHS cohort, only oxygen use was reported more commonly in HIV-infected participants, although oxygen use was uncommon overall (Figure 1b). In regression models, HIV infection was independently associated with greater prevalence of cough (prevalence ratio [PR], 1.11; p = 0.03), dyspnea (PR, 1.25; p = 0.03), wheezing (PR 1.20; p = 0.04), and having any respiratory symptom (PR, 1.11; p = 0.003) in the MACS cohort and associated with wheezing (PR, 1.19; p = 0.02), inhaler use (PR 1.14; p = 0.04), and oxygen use (PR 3.43; p = 0.01) in the WIHS cohort (Table 2).

**Table 2:** Association of HIV status and prevalent pulmonary outcomes for each cohort

	**MACS**		**WIHS**	
	**PR (95% CI) for HIV-infected**	**p-value**	**PR (95% CI) for HIV-infected**	**p-value**
**Symptoms**				
Cough	1.11 (1.01-1.21)	0.03	1.02 (0.95-1.09)	0.61
Dyspnea	1.25 (1.02-1.53)	0.03	1.09 (0.98-1.20)	0.12
Wheeze	1.20 (1.00-1.44)	0.04	1.19 (1.03-1.38)	0.02
Sputum	1.08 (0.97-1.20)	0.18	1.08 (0.98-1.19)	0.11
Any symptom	1.11 (1.04-1.19)	0.003	1.04 (0.99-1.10)	0.12
Inhaler use	1.17 (0.99-1.38)	0.06	1.14 (1.00-1.30)	0.04
Oxygen use	1.12 (0.57-2.19)	0.75	3.43 (1.36-8.66)	0.01
**Diagnostic testing**				
Sputum testing	2.93 (2.24-3.83)	<0.001	2.88 (2.13-3.89)	<0.001
Bronchoscopy	2.73 (1.75-4.28)	<0.001	1.67 (0.94-2.98)	0.08
Chest CT	1.28 (1.11-1.47)	0.001	1.24 (1.00-1.53)	0.05
Pulmonary function tests	1.04 (0.92-1.17)	0.55	0.97 (0.82-1.15)	0.76
Echocardiogram	1.27 (1.11-1.46)	0.001	1.02 (0.83-1.27)	0.82
Polysomnography	1.30 (1.06-1.59)	0.01	1.33 (0.92-1.93)	0.13
**Diagnoses**				
Asthma	1.04 (0.80-1.34)	0.78	1.05 (0.88-1.25)	0.57
Sleep apnea	1.42 (1.10-1.84)	0.01	2.10 (1.31-3.36)	0.002
Any COPD	1.31 (0.80-2.14)	0.28	1.35 (0.82-2.24)	0.24

Models are adjusted for age, race, smoking status, pack-years smoked, intravenous drug use, and other factors noted to be significantly associated with the outcome (p < 0.05) in stepwise regression.

MACS - Multicenter AIDS Cohort Study; WIHS - Women’s Interagency HIV Study; PR - Prevalence ratio; CI - confidence intervals; CT – computed tomography; COPD - chronic obstructive pulmonary disease.

### Diagnostic testing

Patterns of the use of respiratory diagnostic testing varied significantly according to HIV status. Diagnostic tests commonly used to evaluate acute respiratory infections such as sputum testing, bronchoscopy, and chest CT scans were more likely in HIV-infected participants (Figure 1c-d). Despite a greater prevalence of chronic respiratory symptoms in HIV-infected participants, HIV-infected individuals in either cohort were no more likely to have mainstay testing for chronic respiratory disease such as pulmonary function testing (Table 2). HIV-infected participants in MACS were more likely to have an echocardiogram and polysomnography.

### Prevalence of respiratory diagnoses

Obstructive lung diseases were the most commonly reported prevalent diagnosis (Figure 1e,f). In both cohorts, asthma was the most common diagnosis. Asthma prevalence was strikingly high in the WIHS cohort in both HIV-infected (22.9%) and HIV-uninfected (25.7%) participants, but not significantly different by HIV status. COPD diagnoses were also common and mostly attributable to chronic bronchitis, but were no more likely in HIV-infected compared to HIV-uninfected in either cohort.

Sleep apnea appeared to occur at similar prevalence rates in HIV-infected and HIV-uninfected men as well as women, however, when adjusting for confounders such as age and BMI, HIV-infected participants were more likely to have been diagnosed with sleep apnea in both MACS, (PR, 1.42; p = 0.01) and WIHS (PR, 2.10; p = 0.002) (Table 2).

The prevalences were low and did not differ between HIV-infected and HIV-uninfected participants for the diagnoses of pulmonary hypertension (MACS: HIV-infected, 0.6% vs. HIV-uninfected, 0.4%; WIHS: 0.7% vs. 1.0%), interstitial pulmonary fibrosis (MACS: 0% vs. 0.2%; WIHS: 0% vs. 0.2%), sarcoidosis (MACS: 0.4% vs. 0.2%; WIHS: 0.2% vs. 0.9%), and pulmonary embolism (MACS: 0.5% vs. 1.0%; WIHS: 0.4% vs. 0.7%).

### Prevalence by smoking status

When analysis was performed comparing prevalence of symptoms, diagnostic testing, and diagnoses between HIV-infected and HIV-uninfected subjects stratified by smoking, there appeared to be increased prevalence of outcomes with former and current smoking compared to never smokers (Additional file 1: Table S4a and b). Compared to HIV-uninfected never smokers, HIV-infected men who were never smokers were more likely to have dyspnea and have had bronchoscopy and sputum sampling performed. Compared to HIV-uninfected former smokers, HIV-infected men who were former smokers were more likely to have phlegm production, use inhalers, and have had chest CT, bronchoscopy, and sputum sampling performed, and compared to HIV-uninfected current smokers, HIV-infected men who were current smokers were more likely to have had chest CT, echocardiogram, or sputum sampling performed. HIV-infected women were more likely to have sputum sampling done independent of smoking status, but compared to HIV-uninfected former smokers who were women, female HIV-infected former smokers were more likely to have had chest CT. Compared to HIV-uninfected current smoker women, HIV-infected current smokers were more likely to have wheezing, use an inhaler, and be diagnosed with COPD and chronic bronchitis. An interaction between smoking status and HIV infection was not found to be significant for any respiratory symptoms or diagnoses.

### Incidence of respiratory diagnoses

Incidence of diagnoses was determined during 2 years following the prevalence questionnaire. Men had a higher incidence rate of sleep apnea compared to women, while women had higher incidence rates for asthma and COPD compared to men (Table 3). HIV-infected individuals were more likely to report a new diagnosis of COPD in the MACS cohort (rate ratio [RR], 2.21; p = 0.03), and there was a borderline association with HIV and increased incident sleep apnea (RR 2.33; p = 0.08) in the WIHS cohort.

**Table 3:** Estimates for the effect of HIV infection on incidence of pulmonary diagnoses

	**MACS**				**WIHS**			
	**Incident rates per 100 person-years**		** RR (95% CI)**	**p-value**	**Incident rates per 100 person-years**		** RR (95% CI)**	**p-value**
**Diagnosis**	**HIV-infected**	**HIV-uninfected**	**HIV-infected vs. HIV-uninfected**		**HIV-infected**	**HIV-uninfected**	**HIV-infected vs. HIV-uninfected**	
Asthma	0.69	0.62	0.86 (0.35-2.13)	0.75	2.59	3.35	0.87 (0.54-1.41)	0.58
COPD	1.70	0.69	2.21 (1.09-4.50)	0.03	2.13	1.39	1.58 (0.85-2.94)	0.15
Sleep apnea	1.41	1.45	0.92 (0.49-1.73)	0.81	1.12	0.49	2.33 (0.89-6.11)	0.09

Adjusted for age, race, smoking status, and intravenous drug use.

MACS – Multicenter AIDS Cohort Study; WIHS – Women’s Interagency HIV Study; RR– Rate ratio; CI – confidence interval; COPD – chronic obstructive pulmonary disease.

### Relationship of HIV-associated variables and respiratory symptoms, treatments, testing, and diagnoses

HIV-related variables such as nadir CD4 cell count, HIV viral load, and HAART use were also associated with respiratory symptoms, diagnostic testing, and diagnoses (Additional file 1: Table S3a). In HIV-infected participants in both the MACS and WIHS cohorts, diagnostic testing such as sputum and bronchoscopy were more likely in those with lower nadir CD4 cell counts (Additional file 1: Table S3a). In WIHS HIV-infected participants, COPD was more likely with higher plasma HIV RNA levels.

We also included cardiovascular disease and history of bacterial or *Pneumocystis* pneumonia in models (Additional file 1: Table S3b). In MACS, there were associations of cardiovascular disease and history of pneumonia with various respiratory symptoms (cardiovascular disease, bacterial pneumonia), increased likelihood of bronchoscopy (cardiovascular disease, bacterial pneumonia) or sputum induction (bacterial pneumonia, *Pneumocystis* pneumonia), and with diagnoses of asthma (bacterial pneumonia) and COPD (bacterial pneumonia). Lower nadir CD4 counts were no longer associated with increased chance of sputum testing and bronchoscopy. In WIHS, dyspnea was significantly increased in those with cardiovascular disease or a history of bacterial pneumonia with a trend to being more common in those on HAART (Additional file 1: Table S3b). Wheezing was also more common in those with a history of bacterial pneumonia or in those using HAART. Individuals with a history of cardiovascular disease were more likely to have had an echocardiogram and those with bacterial pneumonia or *Pneumocystis* pneumonia were more likely to have had a bronchoscopy of sputum induction. A history of pneumonia was more common in individuals with asthma (bacterial pneumonia) or COPD (bacterial or *Pneumocystis* pneumonia). Higher HIV RNA level was no longer significantly associated with a COPD diagnosis.

## Discussion

This study is the first during the current HAART era of the HIV epidemic to assess respiratory symptoms, diagnostic testing, and a broad number of pulmonary diagnoses and compare them between HIV-infected and HIV-uninfected persons in a large, multicenter study. We found that HIV infection was independently associated with higher prevalence of certain respiratory symptoms, with certain aspects of HIV associated with different respiratory symptoms. Despite an increase in chronic respiratory symptoms, the HIV-infected groups were no more likely to have evaluation of non-infectious pulmonary diseases, suggesting that these diseases may be underdiagnosed in HIV. HIV infection was an independent risk factor for prevalent sleep apnea and incident (in MACS) COPD.

Persons infected with HIV have been noted to have a high burden of respiratory symptoms, both during the pre-HAART [14] and the current HAART eras [17]. Our findings support that HIV infection confers an independent risk for increased respiratory symptoms, and poor HIV control may contribute to the increase in symptoms, although the relationship to various aspects of immunosuppression is likely variable and complex. The impact of respiratory symptoms on quality of life in this population is unclear, but increased respiratory symptom burden and medication use may add significantly to the frailty experienced by patients [27].

As non-infectious pulmonary diseases emerge as important causes of morbidity and mortality in the current era of HIV, our data suggest that these chronic pulmonary disorders are likely under-recognized and therefore under-treated. Despite the increase in symptoms, diagnostic testing in HIV did not reflect the burden of chronic respiratory symptoms. Testing generally used to diagnose infectious complications of HIV (such as sputum testing, bronchoscopy, and CT scans) were more common in HIV, but despite an increase in chronic respiratory symptoms and greater self-report of diagnoses such as COPD and sleep apnea in the HIV-infected groups, there was no increase in testing such as pulmonary function in either cohort or in polysomnography in WIHS that would assess these conditions. This finding may reflect a diagnostic bias which prevents many physicians from evaluating HIV-infected persons for chronic respiratory conditions such as asthma or COPD and suggests that these diseases may actually be more common in HIV than recognized.

Our findings build on prior data that HIV infection is independently associated with COPD in cohorts of veterans or intravenous drug users [1,6,7]. Accelerated emphysema and COPD have long been seen in HIV infection, [28] and recent studies have confirmed this association in the HAART era [1,7]. Our study is a cohort that represents a population different from those in prior studies of veterans [1] or intravenous drug abusers [7]. We also found that higher viral load was associated with a diagnosis of COPD, similar to a recent study finding airflow obstruction associated with viral load greater than 200,000 copies/μL. The prior study controlled for a history of bacterial pneumonia, but when we added a history of cardiovascular disease, bacterial pneumonia, and *Pneumoycstis* pneumonia to the models, these factors were more strongly associated than viral load, suggesting that poorly controlled HIV may be associated with COPD either directly through prior infections or through common pathways linking HIV, cardiovascular disease and COPD [7].

We determined that HIV was significantly associated with sleep apnea, which has not been reported previously in a cohort of this size. Unlike smoking-related diseases such as COPD, [12,29] the most common risk factor for sleep apnea, heavier body mass, [30] was not more common in HIV-infected persons. Yet, HIV-infected persons were more likely to be diagnosed and treated for sleep apnea despite lower average BMI. Prior literature suggests that sleep apnea in HIV may be related less to traditional anthropomorphic risks seen in the general population [31] and may be explained by upper airway abnormalities related to adenoid or tonsilar hypertrophy [32]. Metabolic disease and abnormal adipose distribution related to HIV infection and antiretroviral medications [33] could also play a role, and there was a trend towards association between sleep apnea and antiretroviral use in these cohorts although it did not reach significance. Sleep apnea is associated with cardiovascular disease in the general population [34] and may be an important mediator of increased cardiovascular disease seen in HIV [35].

Associations between HIV status and some respiratory outcomes differed between men and women in this study. While this incongruity may be explained by differences other than sex (proportions of minorities, socioeconomic status, access to health care, and substance use), gender differences in susceptibility to lung disease may also play a role. Rates of asthma are greater in adult women compared to men, [36] and women are more susceptible to airflow obstruction than men given equivalent exposure to cigarette smoking [37,38]. The influence of lung dimensions and hormonal influence on airway inflammation could explain the differences we found in which symptoms and diagnoses were associated with HIV in women compared to men.

The pathogenesis of respiratory disease in HIV-infected persons is not completely understood and may vary by diagnosis. Increased pulmonary and systemic inflammation may play an important role in pulmonary hypertension, [39,40] while in asthma, metabolic disease and Th2 inflammation also have significant associations [41]. Our findings support that, in addition to traditional risk factors for lung disease (smoking and substance abuse), factors related to HIV infection (viremia or immune suppression) play a role in the pathogenesis in HIV-infected persons. Antiretroviral medication has been associated with worse airflow obstruction in HIV infection [18,19]. One proposed mechanism that may explain the association of antiretroviral use and airway obstruction is immune reconstitution causing airway inflammation. Another reason for increased respiratory disease in HIV-infected persons may be residual lung abnormalities from prior infections or colonizing organisms. Recent data shows that HIV-infected persons are still more likely to have bacterial or mycobacterial pneumonias [1]. Studies using culture-independent techniques have found that detection of *Pneumocystis* is associated with airflow obstruction, and this association has been confirmed in animal studies [42-44]. In addition, a recent study of the lung microbiome demonstrated that HIV-infected individuals are more likely to have *Trophyrema whipplei* detected in bronchoscopic alveolar lavage, although the relationship to pulmonary function is not known [45]. Our findings of the relationship of prior pneumonia to respiratory complications suggest that infections and resulting lung damage may be important.

Antiretroviral toxicity may also play a role. Some antiretroviral medications have been linked to central obesity, which has been associated with increased inflammation related to higher leptin and lower adiponectin levels [46,47]. These changes may contribute to increased cardiovascular disease in HIV, [48] and our findings suggest obesity may play a role in certain lung diseases as well as we found that increased body mass index is associated with several symptoms and lung diagnoses in HIV-infected women. In the general population, obesity has increasingly been recognized as a risk for respiratory diseases [49].

Our study is limited in that most data were collected from a cross-sectional assessment, and longitudinal data on outcomes were collected over a period of only two years. Nearly 11% and 19% of men and women, respectively, in the cohorts did not complete the pulmonary survey, with those who had completed being older and more likely to use intravenous drugs and cocaine which could make our prevalence and incidence estimates higher and less generalizable to the HIV population. Also, it may not be possible to directly invoke a specific cardiac or respiratory cause for the symptoms reported, but our findings suggest that both cardiac and respiratory disease could be potential etiologies of symptoms in this population. Additionally, our data were obtained by self-report which could suffer from recall or motivational bias. These limitations are counteracted by having a large number of participants, an HIV-uninfected group that has similar risk for HIV exposure as the HIV-infected participants, and detailed prospective collection of data on covariates over several decades. These cohorts have a high prevalence of smoking and drug use which may limit the application of our findings to other HIV populations, although these risk factors are quite common in HIV-infected persons in the US [10]. Differential rates of various diagnostic testing may also lead to differential rates of diagnosis in certain groups. Additionally, in these cohorts, the HIV-infected groups have been followed for several decades, with many of the originally enrolled HIV-infected participants dying and being repopulated with new recruits prior to our respiratory data collection. This structure may introduce a certain amount of survival bias and has also created a younger HIV-infected group, which we would expect to bias away from finding differences associated with HIV.

## Conclusion

In conclusion, we found that HIV infection remains an important risk factor for respiratory diseases. These data add to the current literature by demonstrating an increased incidence of obstructive lung disease among HIV-infected individuals and by establishing HIV as an independent risk factor for sleep apnea. The lack of testing for chronic respiratory complaints in HIV-infected individuals suggests that the true prevalence and incidence of chronic lung disease may actually be higher than observed. Further, awareness of chronic lung diseases in HIV-infected persons is necessary in order to ensure diagnostic evaluation and appropriate treatment.

## Competing interests

The authors have no competing interests to report related to the manuscript.

## Authors’ contributions

Contributions: Drs. MRG, GKB, MB, and AM had full access to all of the data in the study and take responsibility for the integrity of the data and the accuracy of the data analysis. Study concept and design: MRG, LK, and AM Analysis and interpretation of data: MRG, GKB, TBR, LK, ECK, RD, ECS, RMG, SH, LH, SHS, MB, and AM. Drafting of the manuscript: MRG and AM. Critical revision of the manuscript for important intellectual content: MRG, GKB, TBR, LK, ECK, RD, ECS, RMG, SH, LH, SHS, MB, and AM. Statistical analysis: MRG, GKB, ECS, MB, and AM. Administrative, technical, or material support: MRG. Study supervision:MRG, GKB, LK, ECK, RD, ECS, RMG, SH, LH, SHS, MB, and AM. All authors read and approved the final manuscript.

## Pre-publication history

The pre-publication history for this paper can be accessed here:

http://www.biomedcentral.com/1471-2466/14/75/prepub

## Supplementary Material

Additional file 1: Table S1 Comparison of participants who completed the pulmonary related questionnaire vs. participants enrolled in the MACS and WIHS studies who did not complete the questionnaire. **Table S2.** Proportion of participants with prevalent symptoms, diagnostic testing, and diagnoses comparing HIV-infected and HIV-uninfected by cohort. **Table S3a.** Final adjusted Poisson regression results for factors associated with prevalent pulmonary outcomes in the HIV-infected subgroup for each cohort. **Table S3b.** Final adjusted Poisson regression results for factors associated with prevalent pulmonary outcomes in the HIV-infected subgroup for each cohort including cardiovascular disease and history of bacterial or *Pneumocystis* pneumonia in variable selection. **Table S4a.** Proportion of patients with prevalent symptoms, diagnostic testing, and diagnoses comparing HIV-infected and HIV-uninfected by smoking status - MACS. **Table S4b.** Proportion of patients with prevalent symptoms, diagnostic testing, and diagnoses comparing HIV-infected and HIV-uninfected by smoking status - WIHS. **Table S5a.** Final adjusted Poisson regression models of baseline factors associated with prevalent pulmonary outcomes in the MACS cohort. **Table S5b.** Final adjusted Poisson regression models of factors associated with prevalent pulmonary outcomes in the WIHS cohort. Click here for file
