# Supplementary material for: Pulmonary symptoms and diagnoses are associated with HIV in the MACS and WIHS cohorts
Source: BMC Pulm Med. 2014 Apr 30;14:75. doi: 10.1186/1471-2466-14-75 (PMC4021087; doi:10.1186/1471-2466-14-75)
Supplement: Additional file 1: Table S1 — Comparison of participants who completed the pulmonary related questionnaire vs. participants enrolled in the MACS and WIHS studies who did not complete the questionnaire. Table S2. Proportion of participants with prevalent symptoms, diagnostic testing, and diagnoses comparing HIV-infected and HIV-uninfected by cohort. Table S3a. Final adjusted Poisson regression results for factors associated with prevalent pulmonary outcomes in the HIV-infected subgroup for each cohort. Table S3b. Final adjusted Poisson regression results for factors associated with prevalent pulmonary outcomes in the HIV-infected subgroup for each cohort including cardiovascular disease and history of bacterial or Pneumocystis pneumonia in variable selection. Table S4a. Proportion of patients with prevalent symptoms, diagnostic testing, and diagnoses comparing HIV-infected and HIV-uninfected by smoking status - MACS. Table S4b. Proportion of patients with prevalent symptoms, diagnostic testing, and diagnoses comparing HIV-infected and HIV-uninfected by smoking status - WIHS. Table S5a. Final adjusted Poisson regression models of baseline factors associated with prevalent pulmonary outcomes in the MACS cohort. Table S5b. Final adjusted Poisson regression models of factors associated with prevalent pulmonary outcomes in the WIHS cohort. [file 1471-2466-14-75-S1.docx]

Supplemental material

**Pulmonary symptoms and diagnoses are associated with HIV in the MACS and WIHS cohorts**

Matthew R. Gingo MD, MS^1^, G. K. Balasubramani PhD^2^, Thomas B. Rice MD, MS^1^, Lawrence Kingsley DrPH^2,3^, Eric C. Kleerup MD^4^, Roger Detels MD^4^, Eric Seaberg PhD, MPH^5^, Ruth M. Greenblatt MD^6,7,8^, Susan Holman RN, MS^9^, Laurence Huang MD, MAS^7^, Sarah H. Sutton MD^10^, Marnie Bertolet, PhD^2^, Alison Morris MD, MS^1,11^

^1^Department of Medicine, School of Medicine, University of Pittsburgh, Pittsburgh, PA, USA

^2^Epidemiology Data Center, Department of Epidemiology, School of Public Health, University of Pittsburgh, Pittsburgh, PA, USA

^3^Department of Infectious Diseases and Microbiology, School of Public Health, University of Pittsburgh, Pittsburgh, PA, USA

^4^Department of Medicine, David Geffen School of Medicine, University of California, Los Angeles, CA, USA

^5^Department of Epidemiology, Bloomberg School of Public Health, Johns Hopkins University, Baltimore, MD, USA

^6^Department of Clinical Pharmacy, School of Pharmacy, University of California, San Francisco, CA, USA

^7^Department of Medicine, School of Medicine, University of California, San Francisco, CA, USA

^8^Department of Epidemiology and Biostatistics, School of Medicine, University of California, San Francisco, CA, USA

^9^Department of Medicine, SUNY Downstate Medical Center, Brooklyn, NY, USA

^10^Department of Medicine, Feinberg School of Medicine, Northwestern University, Chicago, IL, USA

^11^Department of Immunology, School of Medicine, University of Pittsburgh, PA, USA

Additional file 1: Table S1: Comparison of participants who completed the pulmonary related questionnaire vs. participants enrolled in the MACS and WIHS studies who did not complete the questionnaire.

|  | **MACS** |  |  | **WIHS** |  |  |
| --- | --- | --- | --- | --- | --- | --- |
| **Questionnaire complete?** | **Yes (n=1896)** | **No (n=223)** | **p-value** | **Yes (n=1976)** | **No (n=488)** | **p-value** |
| HIV-infected, n (%) | 907 (47.8) | 102 (45.7) | 0.55 | 1405 (71.1) | 357 (73.2) | 0.37 |
| Age, mean (SD) | 50.8 (9.9) | 34.5 (8.2) | <0.001 | 43.4 (9.3) | 39.8 (9.2) | <0.001 |
| African American, n (%) | 482 (25.4) | 74 (33.2) | 0.01 | 1183 (59.9) | 319 (66.1) | 0.01 |
| Hispanic, n (%) | 170 (9.0) | 27 (12.1) | 0.13 | 541 (27.4) | 86 (17.8) | <0.001 |
| Smoking Status, n (%) |  |  | 0.43 |  |  | 0.003 |
| Never | 480 (25.6) | 51 (27.3) |  | 577 (29.5) | 166 (34.9) |  |
| Former | 894 (47.7) | 80 (42.8) |  | 543 (27.8) | 98 (20.6) |  |
| Current | 501 (26.7) | 56 (29.9) |  | 835 (42.7) | 212 (44.5) |  |
| Pack year-Median (Q1-Q3) | 2.2 (0-21) | 4.3 (0-21) | 0.27 | 13 (0-25.9) | 13 (0-26) | 0.72 |
| Alcohol use, n (%) |  |  | 0.83 |  |  | 0.002 |
| None | 356 (19.0) | 39 (21.7) |  | 1170 (59.9) | 242 (50.8) |  |
| Light | 940 (50.2) | 86 (47.8) |  | 529 (27.1) | 149 (31.3) |  |
| Moderate | 429 (22.9) | 42 (23.3) |  | 184 (9.4) | 56 (11.8) |  |
| Heavy | 147 (7.9) | 13 (7.2) |  | 72 (3.6) | 29 (6.1) |  |
| Intravenous drug use-ever, n (%) | 233 (12.3) | 3 (1.7) | <0.001 | 495 (25.1) | 11 (2.3) | <0.001 |
| Cocaine use-ever, n (%) | 947 (49.9) | 11 (6.2) | <0.001 | 1102 (55.8) | 14 (2.9) | <0.001 |
|  |  |  |  |  |  |  |
| HIV POSITIVE ONLY |  |  |  |  |  |  |
| CD4 count (cells/µL)-current, mean (SD) | 571.8 (277.8) | 516.8 (252.4) | 0.29 | 502 (291.2) | 529.6 (287.7) | 0.12 |
| Plasma HIV RNA level (copies/mL), median (Q1-Q3) | 40 (40-136) | 40 (40-14900) | <0.001 | 80 (80-2400) | 80 (48-2430) | <0.001 |
| Plasma HIV RNA undetectable, n (%)* | 590 (70.0) | 42 (51.9) | 0.001 | 764 (56.0) | 185 (54.6) | 0.64 |

* HIV RNA level at baseline pulmonary questionnaire visit available in 924 MACS (843 who completed the questionnaire, 81 who did not complete the questionnaire) and 1704 WIHS participants (1365 who completed the questionnaire, 339 who did not complete the questionnaire). Undetectable level was <40 copies/mL in the MACS cohort and <80 copies/mL in the WIHS cohort.

MACS - Multicenter AIDS Cohort Study; WIHS - Women’s Interagency HIV Study; SD - standard deviation; n - number; % - percentage; Q1 - quartile 1; Q3 - quartile 3

Additional file 1: Table S2. Proportion of participants with prevalent symptoms, diagnostic testing, and diagnoses comparing HIV-infected and HIV-uninfected by cohort.

|  | **MACS** |  |  | **WIHS** |  |  |
| --- | --- | --- | --- | --- | --- | --- |
|  | **HIV-uninfected** | **HIV-infected** |  | **HIV-uninfected** | **HIV-infected** |  |
|  | **n=989** | **n=907** | **p-value** | **n=571** | **n=1405** | **p-value** |
| **Symptoms** |  |  |  |  |  |  |
| Cough, n (%) | 372 (38.6) | 377 (42.6) | 0.08 | 332 (58.2) | 758 (54.2) | 0.10 |
| Dyspnea, n (%) | 93 (9.4) | 123 (13.8) | 0.003 | 209 (36.7) | 508 (36.3) | 0.88 |
| Wheezing, n (%) | 127 (12.9) | 140 (15.6) | 0.10 | 127 (22.3) | 343 (24.6) | 0.28 |
| Phlegm production, n (%) | 291 (29.8) | 314 (34.9) | 0.02 | 222 (39.0) | 570 (40.8) | 0.47 |
| Any symptom, n (%) | 479 (49.5) | 497 (56.2) | 0.004 | 385 (67.7) | 926 (66.3) | 0.56 |
| Inhaler use, n (%) | 218 (22.1) | 247 (27.4) | 0.008 | 212 (37.2) | 559 (39.9) | 0.25 |
| Oxygen use, n (%) | 10 (1.0) | 16 (1.8) | 0.16 | 6 (1.1) | 34 (2.4) | 0.049 |
| **Diagnostic** **testing** |  |  |  |  |  |  |
| Chest computed tomography, n (%) | 224 (23.7) | 294 (34.4) | <0.001 | 71 (12.5) | 224 (16.1) | 0.04 |
| Bronchoscopy, n (%) | 27 (2.8) | 59 (6.6) | <0.001 | 14 (2.5) | 56 (4.0) | 0.09 |
| Pulmonary function tests, n (%) | 368 (39.1) | 340 (38.6) | 0.84 | 152 (26.7) | 366 (26.2) | 0.81 |
| Lung surgery, n (%) | 10 (1.0) | 16 (1.8) | 0.16 | 6 (1.1) | 16 (1.1) | 0.97 |
| Sputum sample, n (%) | 52 (5.4) | 154 (17.3) | <0.001 | 39 (6.8) | 284 (20.3) | <0.001 |
| Polysomnography, n (%) | 152 (15.5) | 155 (17.2) | 0.33 | 37 (6.5) | 83 (5.9) | 0.64 |
| Echocardiogram, n (%) | 275 (29.0) | 259 (29.4) | 0.84 | 87 (15.3) | 222 (15.9) | 0.72 |
| **Diagnoses** |  |  |  |  |  |  |
| Asthma, n (%) | 120 (12.2) | 122 (13.5) | 0.38 | 146 (25.7) | 320 (22.9) | 0.18 |
| COPD, n (%) | 66 (6.7) | 73 (8.1) | 0.25 | 59 (10.4) | 215 (15.4) | 0.004 |
| Chronic bronchitis, n (%) | 53 (5.4) | 57 (6.3) | 0.39 | 54 (9.5) | 182 (13.0) | 0.03 |
| Sleep apnea | 107 (10.8) | 94 (10.5) | 0.79 | 16 (2.8) | 60 (4.3) | 0.12 |
| Pulmonary hypertension | 6 (0.6) | 4 (0.4) | 0.62 | 4 (0.7) | 14 (1.0) | 0.53 |
| Interstitial pulmonary fibrosis, n (%) | - | 2 (0.2) | - | - | 3 (0.2) | - |
| Sarcoidosis, n (%) | 4 (0.4) | 2 (0.2) | 0.48 | 1 (0.2) | 13 (0.9) | 0.07 |
| Pulmonary embolism, n (%) | 5 (0.5) | 9 (1.0) | 0.21 | 2 (0.4) | 10 (0.7) | 0.35 |

COPD - chronic obstructive pulmonary disease.

Additional file 1: Table S3a. Final adjusted Poisson regression results for factors associated with prevalent pulmonary outcomes in the HIV-infected subgroup for each cohort.

|  |  | **MACS** |  | **WIHS** |  |
| --- | --- | --- | --- | --- | --- |
|  |  | **Adjusted PR (95%CI)** | **p-value** | **Adjusted PR (95%CI)** | **p-value** |
| **Outcomes** | **Independent Variable** |  |  |  |  |
| **Symptoms** |  |  |  |  |  |
| Cough | HAART use (ref=Never) |  | 0.14 |  | 0.18 |
|  | Past | 1.69 (0.97-2.95) |  | 0.96 (0.71-1.29) |  |
|  | Current | 1.37 (0.80-2.36) |  | 0.81 (0.61-1.07) |  |
|  | Smoking (ref=Never) |  | <0.001 |  |  |
|  | Current | 1.78 (1.26-2.50) |  |  |  |
|  | Former | 1.02 (0.75-1.38) |  |  |  |
|  |  |  |  |  |  |
| Dyspnea | HAART use (ref=Never) |  | 0.17 |  | 0.23 |
|  | Past | 2.25 (0.76-6.66) |  | 1.24 (0.84-1.82) |  |
|  | Current | 1.47 (0.50-4.29) |  | 1.00 (0.69-1.45) |  |
|  | Cocaine use, ever |  |  | 1.37 (1.06-1.77) | 0.01 |
|  | BMI (per 3 units) |  |  | 1.05 (1.01-1.08) | 0.01 |
|  | Age (per 5 Years) | 0.84 (0.75-0.95) | 0.004 |  |  |
|  |  |  |  |  |  |
| Wheeze | HAART use (ref=Never) |  | 0.39 |  | 0.09 |
|  | Past | 2.04 (0.68-6.10) |  | 1.59 (0.98-2.58) |  |
|  | Current | 1.57 (0.54-4.59) |  | 1.23 (0.76-1.97) |  |
|  | Smoking (ref=Never) |  | 0.05 |  | 0.001 |
|  | Current | 1.66 (0.89-3.11) |  | 1.85 (1.16-2.96) |  |
|  | Former | 0.98 (0.56-1.73) |  | 1.07 (0.71-1.63) |  |
|  | Cocaine use, ever | 1.60 (1.06-2.42) | 0.03 | 1.43 (1.05-1.96) | 0.02 |
|  | Smoking (per 10 pack years) | 1.14 (1.04-1.25) | 0.007 |  |  |
|  | BMI (per 3 units) |  |  | 1.07 (1.03-1.12) | 0.001 |
|  |  |  |  |  |  |
| Sputum | HAART use (ref=Never) |  | 0.53 |  | 0.47 |
|  | Past | 1.29 (0.71-2.35) |  | 0.94 (0.66-1.34) |  |
|  | Current | 1.07 (0.60-1.91) |  | 0.84 (0.60-1.17) |  |
|  | Smoking (ref=Never) |  | 0.01 |  | 0.01 |
|  | Current | 1.55 (1.07-2.25) |  | 1.71 (1.21-2.41) |  |
|  | Former | 1.00 (0.72-1.39) |  | 1.33 (1.00-1.78) |  |
|  |  |  |  |  |  |
| Any symptom | HAART use (ref=Never) |  | 0.31 |  | 0.48 |
|  | Past | 1.42 (0.88-2.30) |  | 0.96 (0.72-1.27) |  |
|  | Current | 1.23 (0.77-1.96) |  | 0.87 (0.67-1.14) |  |
|  | Smoking (ref=Never) |  | 0.01 |  | 0.03 |
|  | Current | 1.45 (1.08-1.95) |  | 1.40 (1.07-1.83) |  |
|  | Former | 1.03 (0.79-1.33) |  | 1.14 (0.91-1.43) |  |
|  | BMI (per 3 units) |  |  | 1.03 (1.00-1.06) | 0.03 |
|  |  |  |  |  |  |
| Inhaler use | HAART use (ref=Never) |  | 0.57 |  | 0.66 |
|  | Past | 1.44 (0.71-2.91) |  | 1.03 (0.70-1.51) |  |
|  | Current | 1.24 (0.63-2.44) |  | 1.14 (0.79-1.63) |  |
|  | Smoking (ref=Never) |  |  |  | 0.03 |
|  | Current |  |  | 1.52 (1.06-2.18) |  |
|  | Former |  |  | 1.16 (0.85-1.58) |  |
|  | Cocaine use, ever |  |  | 1.51 (1.19-1.93) | 0.001 |
|  | BMI (per 3 units) |  |  | 1.06 (1.03-1.10) | 0.002 |
|  |  |  |  |  |  |
| Oxygen use **^1^** | HAART use (ref=Never) |  | 0.13 |  | 0.78 |
|  | Past |  |  | 1.01 (0.69-1.47) |  |
|  | Current | 0.34 (0.09-1.36) |  | 1.09 (0.77-1.55) |  |
|  | Smoking (ref=Never) |  |  |  | 0.01 |
|  | Current |  |  | 1.72 (1.21-2.45) |  |
|  | Former |  |  | 1.35 (1.01-1.82) |  |
|  | IDU, ever |  |  | 1.23 (1.00-1.51) | 0.05 |
|  | Nadir in CD4 count (per 200 counts) | 0.30 (0.11-0.81) | 0.02 |  |  |
| **Diagnostic testing** |  |  |  |  |  |
| Chest CT | HAART use (ref=Never) |  | 0.48 |  | 0.72 |
|  | Past | 1.44 (0.74-2.80) |  | 0.95 (0.50-1.81) |  |
|  | Current | 1.19 (0.63-2.26) |  | 1.13 (0.63-2.03) |  |
|  | IDU, ever |  |  | 1.40 (1.01-1.94) | 0.04 |
|  | Age (per 5 Years) |  |  | 1.11 (1.01-1.22) | 0.03 |
|  |  |  |  |  |  |
| Echocardiogram | HAART use (ref=Never) |  | 0.34 |  | 0.03 |
|  | Past | 1.43 (0.72-2.83) |  | 0.54 (0.28-1.06) |  |
|  | Current | 1.06 (0.55-2.04) |  | 1.10 (0.63-1.92) |  |
|  | Black | 0.69 (0.50-0.96) | 0.03 |  |  |
|  | BMI (per 3 units) |  |  | 1.08 (1.03-1.14) | 0.002 |
|  | Age (per 5 Years) | 1.15 (1.06-1.25) | 0.001 | 1.23 (1.13-1.34) | <0.001 |
|  |  |  |  |  |  |
| Bronchoscopy **^1^** | HAART use (ref=Never) |  | 0.47 |  | 0.29 |
|  | Past |  |  | 0.64 (0.11-3.58) |  |
|  | Current | 1.43 (0.54-3.78) |  | 1.50 (0.34-6.68 |  |
|  | Black | 1.79 (1.00-3.23) | 0.05 |  |  |
|  | Nadir CD4 count (per 200 counts) | 0.43 (0.28-0.67) | 0.002 | 0.58 (0.36-0.92) | 0.02 |
|  |  |  |  |  |  |
| Polysomnography | HAART use (ref=Never) |  | 0.91 |  | 0.80 |
|  | Past | 1.11 (0.51-2.43) |  | 0.72 (0.27-1.94) |  |
|  | Current | 0.98 (0.47-2.06) |  | 0.86 (0.36-2.08) |  |
|  | Black | 1.49 (1.02-2.16) | 0.04 |  |  |
|  | Hispanic | 0.31 (0.11-0.85) | 0.02 | 0.10 (0.03-0.29) | <0.001 |
|  | BMI (per 3 units) | 1.17 (1.06-1.30) | 0.002 | 1.15 (1.07-1.23) | 0.0002 |
|  | Age (per 5 Years) | 1.20 (1.07-1.34) | 0.002 |  |  |
|  |  |  |  |  |  |
| Pulmonary function tests | HAART use (ref=Never) |  | 0.43 |  | 0.99 |
|  | Past | 0.91 (0.50-1.65) |  | 0.98 (0.62-1.56) |  |
|  | Current | 1.19 (0.68-2.07) |  | 1.00 (0.65-1.54) |  |
|  | Cocaine use, ever |  |  | 1.52 (1.13-2.03) | 0.01 |
|  | Age (per 5 Years) |  |  | 1.10 (1.02-1.18) | 0.01 |
|  |  |  |  |  |  |
| Sputum testing | HAART use (ref=Never) |  | 0.49 |  | 0.22 |
|  | Past | 1.07 (0.45-2.56) |  | 0.73 (0.41-1.31) |  |
|  | Current | 0.79 (0.34-1.84) |  | 1.05 (0.63-1.75) |  |
|  | Smoking (ref=Never) |  |  |  | 0.04 |
|  | Current |  |  | 1.99 (1.15-3.45) |  |
|  | Former |  |  | 1.51 (0.94-2.43) |  |
|  | Cocaine use, ever |  |  | 1.48 (1.04-2.12) | 0.03 |
|  | Nadir CD4 count (per 200 counts) | 0.67 (0.53-0.86) | 0.001 | 0.81 (0.67-0.98) | 0.03 |
|  | Age (per 5 Years) |  |  | 1.14 (1.04-1.24) | 0.004 |
| **Diagnoses** |  |  |  |  |  |
| Asthma | HAART use (ref=Never) |  | 0.87 |  | 0.61 |
|  | Past | 0.85 (0.36-1.97) |  | 1.19 (0.72-1.95) |  |
|  | Current | 0.99 (0.44-2.20) |  | 1.02 (0.63-1.64) |  |
|  | Smoking (ref=Never) |  |  |  | 0.05 |
|  | Current |  |  | 1.63 (1.01-2.64) |  |
|  | Former |  |  | 1.18 (0.77-1.79) |  |
|  | Cocaine use, ever |  |  | 1.68 (1.22-2.31) | 0.001 |
|  | BMI (per 3 units) |  |  | 1.08 (1.03-1.12) | 0.001 |
|  | Age (per 5 Years) | 0.89 (0.80-1.00) | 0.05 |  |  |
|  |  |  |  |  |  |
| Any COPD | HAART use (ref=Never) |  | 0.93 |  | 0.54 |
|  | Past | 0.80 (0.23-2.84) |  | 0.78 (0.43-1.42) |  |
|  | Current | 0.92 (0.29-2.94) |  | 0.98 (0.57-1.69) |  |
|  | Cocaine use, ever |  |  | 2.09 (1.37-3.21) | 0.001 |
|  | Viral Load |  |  | 1.07 (1.01-1.14) | 0.02 |
|  | Smoking (per 10 pack years) |  |  | 1.23 (1.03-1.47) | 0.02 |
|  |  |  |  |  |  |
| Sleep apnea | HAART use (ref=Never) |  | 0.25 |  | 0.28 |
|  | Past | 2.50 (0.66-9.45) |  | 2.09 (0.53-8.19) |  |
|  | Current | 1.51 (0.42-5.44) |  | 2.81 (0.77-10.3) |  |
|  | BMI (per 3 units) | 1.42 (1.26-1.59) | <0.001 | 1.22 (1.13-1.32) | <0.001 |
|  | Age (per 5 Years) | 1.29 (1.13-1.48) | 0.0002 |  |  |
|  |  |  |  |  |  |
| Pulmonary hypertension **^2^** | HAART use (ref=Never/Past) |  | 0.63 |  | 0.15 |
|  | Current | 2.18 (0.09-52.1) |  | 3.35 (0.64-17.6) |  |
|  | Viral Load | 1.60 (1.07-2.41) | 0.02 |  |  |
|  | Nadir CD4 count (per 200 counts) | 3.95 (1.19-13.1) | 0.03 |  |  |

Models controlled for age, race, smoking status, pack-years smoked, and intravenous drug use.

A variable with ‘blank’ indicates not significant.

**^1^** MACS-Oxygen use/Bronchoscopy - HAART use was classified into two groups only: Never/Past vs. Current, because there were zero frequencies in one of the groups.

**^2^** Pulmonary hypertension - HAART and smoking status were classified into two groups only: Never/former vs. Current for MACS and WIHS.

MACS - Multicenter AIDS Cohort Study; WIHS - Women’s Interagency HIV Study; PR - Prevalent ratio; CI - confidence intervals; HAART – highly active antiretroviral therapy; BMI – body mass index; ICU – injection drug use; COPD - chronic obstructive pulmonary disease.

Additional file 1: Table S3b: Final adjusted Poisson regression results for factors associated with prevalent pulmonary outcomes in the HIV-infected subgroup for each cohort including cardiovascular disease and history of bacterial or *Pneumocystis* pneumonia in variable selection.

|  |  | **MACS** |  | **WIHS** |  |
| --- | --- | --- | --- | --- | --- |
|  |  | **Adjusted PR (95%CI)** | **p-value** | **Adjusted PR (95%CI)** | **p-value** |
| **Outcomes** | **Independent Variable** |  |  |  |  |
| **Symptoms** |  |  |  |  |  |
| Cough | HAART use (ref=Never) |  | 0.14 |  | 0.17 |
|  | Past | 1.69 (0.97-2.95) |  | 0.96 (0.71-1.30) |  |
|  | Current | 1.36 (0.79-2.34) |  | 0.81 (0.60-1.07) |  |
|  | Smoking (ref=Never) |  | <0.001 |  |  |
|  | Current | 1.82 (1.29-2.56 |  |  |  |
|  | Former | 1.03 (0.76-1.39) |  |  |  |
|  |  |  |  |  |  |
| Dyspnea | HAART use (ref=Never) |  | 0.19 |  | 0.07 |
|  | Past | 2.17 (0.73-6.41) |  | 1.31 (0.89-1.94) |  |
|  | Current | 1.44 (0.49-4.21) |  | 0.98 (0.67-1.42) |  |
|  | Cocaine use, ever |  |  | 1.34 (1.04-1.73) | 0.02 |
|  | BMI (per 3 units) |  |  | 1.04 (1.01-1.08) | 0.02 |
|  | Age (per 5 Years) | 0.84 (0.75-0.95) | 0.004 |  |  |
|  | Cardiovascular disease |  |  | 1.49 (1.03-2.18) | 0.04 |
|  | Bacterial pneumonia, ever |  |  | 1.34 (1.08-1.67) | 0.008 |
|  |  |  |  |  |  |
| Wheeze | HAART use (ref=Never) |  | 0.39 |  | 0.04 |
|  | Past | 1.96 (0.66-5.88) |  | 1.69 (1.04-2.75) |  |
|  | Current | 1.49 (0.51-4.37) |  | 1.22 (0.76-1.96) |  |
|  | Smoking (ref=Never) |  | 0.04 |  | 0.001 |
|  | Current | 1.75 (0.94-3.26) |  | 1.86 (1.17-2.97) |  |
|  | Former | 1.00 (0.57-1.77) |  | 1.09 (0.72-0.65) |  |
|  | Cocaine use, ever | 1.63 (1.08-2.46) | 0.02 | 1.41 (1.03-1.93) | 0.03 |
|  | BMI (per 3 units) |  |  | 1.07 (1.03-1.12) | 0.001 |
|  | Cardiovascular disease | 2.12 (1.33-3.40 | 0.002 |  |  |
|  | Bacterial pneumonia, ever | 1.59 (1.03-2.47) | 0.04 | 1.31 (1.01-1.71) | 0.04 |
|  |  |  |  |  |  |
| Sputum | HAART use (ref=Never) |  | 0.53 |  | 0.43 |
|  | Past | 1.28 (0.70-2.33) |  | 0.96 (0.67-1.36) |  |
|  | Current | 1.06 (0.59-1.89) |  | 0.84 (0.60-1.17) |  |
|  | Smoking (ref=Never) |  | 0.01 |  | 0.01 |
|  | Current | 1.57 (1.08-2.29) |  | 1.71 (1.22-2.41) |  |
|  | Former | 1.01 (0.73-1.40) |  | 1.33 (1.00-1.78) |  |
|  | Cardiovascular disease | 1.41 (1.00-2.02) | 0.05 |  |  |
|  |  |  |  |  |  |
| Any symptom | HAART use (ref=Never) |  | 0.31 |  | 0.43 |
|  | Past | 1.42 (0.88-2.30) |  | 0.97 (0.73-1.29) |  |
|  | Current | 1.22 (0.77-1.95) |  | 0.87 (0.67-1.14) |  |
|  | Smoking (ref=Never) |  | 0.01 |  | 0.03 |
|  | Current | 1.48 (1.10-1.99) |  | 1.40 (1.07-1.83) |  |
|  | Former | 1.03 (0.80-1.34) |  | 1.15 (0.92-1.43) |  |
|  | BMI (per 3 units) |  |  | 1.03 (1.00-1.05) | 0.04 |
|  |  |  |  |  |  |
| Inhaler use | HAART use (ref=Never) |  | 0.63 |  | 0.81 |
|  | Past | 1.39 (0.69-2.81) |  | 1.09 (0.74-1.61) |  |
|  | Current | 1.22 (0.62-2.40) |  | 1.12 (0.78-1.61) |  |
|  | Smoking (ref=Never) |  |  |  | 0.03 |
|  | Current |  |  | 1.52 (1.06-2.19) |  |
|  | Former |  |  | 1.17 (0.85-1.59) |  |
|  | Cocaine use, ever |  |  | 1.47 (1.15-1.88) | 0.002 |
|  | BMI (per 3 units) |  |  | 1.06 (1.03-1.10) | 0.0004 |
|  | Age (per 5 Years) | 0.90 (0.83-0.98) | 0.01 |  |  |
|  | Cardiovascular disease | 1.63 (1.10-2.40) | 0.01 |  |  |
|  | Bacterial pneumonia, ever | 1.88 (1.34-2.63) | <0.001 | 1.43 (1.16-1.75) | 0.001 |
|  |  |  |  |  |  |
| Oxygen use **^1^** | HAART use (ref=Never) |  | 0.04 |  | 0.92 |
|  | Past |  |  | 1.07 (0.73-1.56) |  |
|  | Current | 0.20 (0.05-0.92) |  | 1.07 (0.76-1.52) |  |
|  | Smoking (ref=Never) |  |  |  | 0.01 |
|  | Current |  |  | 1.71 (1.20-2.43) |  |
|  | Former |  |  | 1.35 (1.00-1.82) |  |
|  | Smoking (per 10 pack years) | 1.37 (1.02-1.84) | 0.03 |  |  |
|  | Bacterial pneumonia, ever |  |  | 1.50 (1.22-1.84) | <.001 |
|  | *Pneumocystis*, ever | 8.07 (1.77-36.8) | 0.01 |  |  |
|  |  |  |  |  |  |
| **Diagnostic testing** |  |  |  |  |  |
| Chest CT | HAART use (ref=Never) |  | 0.47 |  | 0.96 |
|  | Past | 1.43 (0.74-2.79) |  | 1.06 (0.56-2.01) |  |
|  | Current | 1.18 (0.62-2.24) |  | 1.08 (0.60-1.93) |  |
|  | Bacterial pneumonia, ever |  |  | 1.42 (1.03-1.97) | 0.03 |
|  | *Pneumocystis*, ever |  |  | 1.92 (1.35-2.73) | <0.001 |
| Echocardiogram | HAART use (ref=Never) |  | 0.28 |  | 0.05 |
|  | Past | 1.42 (0.72-2.82) |  | 0.058 (0.30-1.13) |  |
|  | Current | 1.02 (0.53-1.98) |  | 1.11 (0.64-1.92) |  |
|  | Black | 0.69 (0.50-0.96) | 0.03 |  |  |
|  | BMI (per 3 units) |  |  | 1.08 (1.02-1.13) | 0.004 |
|  | Age (per 5 Years) | 1.13 (1.03-1.22) | 0.01 | 1.21 (1.11-1.32) | <0.001 |
|  | Cardiovascular disease | 1.82 (1.27-2.61) | 0.001 | 2.51 (1.57-4.02) | <0.001 |
|  |  |  |  |  |  |
| Bronchoscopy **^1^** | HAART use (ref=Never) |  | 0.45 |  | 0.49 |
|  | Past |  |  | 0.75 (0.13-4.31) |  |
|  | Current | 1.47 (0.53-4.04) |  | 1.44 (0.32-6.44) |  |
|  | Black | 1.99 (1.08-3.66) | 0.03 |  |  |
|  | Nadir CD4 count (per 200 counts) | 0.51 (0.33-0.80) | 0.004 |  |  |
|  | Cardiovascular disease | 2.93 (1.51-5.68) | 0.001 |  |  |
|  | Bacterial pneumonia, ever | 2.28 (1.22-4.25) | 0.01 | 2.71 (1.44-5.07) | 0.002 |
|  | *Pneumocystis*, ever |  |  | 2.62 (1.37-5.02) | 0.004 |
|  |  |  |  |  |  |
| Polysomnography | HAART use (ref=Never) |  | 0.93 |  | 0.83 |
|  | Past | 1.09 (0.50-2.40) |  | 0.73 (0.27-1.99) |  |
|  | Current | 0.98 (0.47-2.05) |  | 0.83 (0.35-1.99) |  |
|  | Black | 1.50 (1.03-2.18) | 0.04 |  |  |
|  | Hispanic | 0.31 (0.11-0.85) | 0.02 | 0.10 (0.03-0.29) | <0.001 |
|  | BMI (per 3 units) | 1.18 (1.06-1.30) | 0.002 | 1.14 (1.06-1.23) | <0.001 |
|  | Age (per 5 Years) | 1.20 (1.07-1.35) | 0.002 |  |  |
|  | Cardiovascular disease |  |  | 3.16 (1.48-6.78) | 0.003 |
|  |  |  |  |  |  |
| Pulmonary function tests | HAART use (ref=Never) |  | 0.42 |  | 0.84 |
|  | Past | 0.90 (0.50-1.64) |  | 1.08 (0.68-1.72) |  |
|  | Current | 1.19 (0.68-2.07) |  | 0.98 (0.64-1.50) |  |
|  | Cocaine use, ever |  |  | 1.44 (1.08-1.94) | 0.01 |
|  | Age (per 5 Years) |  |  | 1.08 (1.00-1.16) | 0.04 |
|  | Bacterial pneumonia, ever | 1.44 (1.06-1.95) | 0.02 | 1.72 (1.34-2.21) | <0.001 |
|  |  |  |  |  |  |
| Sputum testing | HAART use (ref=Never) |  | 0.52 |  | 0.73 |
|  | Past | 1.03 (0.43-2.47) |  | 0.86 (0.48-1.54) |  |
|  | Current | 0.76 (0.32-1.79) |  | 1.01 (0.61-1.69) |  |
|  | Smoking (ref=Never) |  |  |  | 0.04 |
|  | Current |  |  | 2.00 (1.15-3.47) |  |
|  | Former |  |  | 1.58 (0.98-2.54) |  |
|  | Age (per 5 Years) |  |  | 1.11 (1.02-1.22) | 0.02 |
|  | Bacterial pneumonia, ever | 2.30 (1.56-3.40) | <0.001 | 1.98 (1.51-2.59) | <0.001 |
|  | *Pneumocystis*, ever | 2.46 (1.42-4.23) | 0.001 | 1.55 (1.14-2.11) | 0.01 |
| **Diagnoses** |  |  |  |  |  |
| Asthma | HAART use (ref=Never) |  | 0.75 |  | 0.30 |
|  | Past | 0.79 (0.34-1.84) |  | 1.32 (0.80-2.17) |  |
|  | Current | 0.96 (0.43-2.15) |  | 1.03 (0.65-1.65) |  |
|  | Smoking (ref=Never) |  |  |  | 0.05 |
|  | Current |  |  | 1.63 (1.01-2.65) |  |
|  | Former |  |  | 1.19 (0.78-1.81) |  |
|  | Cocaine use, ever |  |  | 1.62 (1.18-2.24) | 0.003 |
|  | BMI (per 3 units) |  |  | 1.07 (1.03-1.12) | 0.001 |
|  | Age (per 5 Years) | 0.88 (0.78-0.98) | 0.02 |  |  |
|  | Bacterial pneumonia, ever | 2.07 (1.26-3.38) | 0.004 | 1.55 (1.19-2.02) | 0.001 |
|  |  |  |  |  |  |
| Any COPD | HAART use (ref=Never) |  | 0.91 |  | 0.97 |
|  | Past | 0.76 (0.21-2.68) |  | 0.93 (0.51-1.69) |  |
|  | Current | 0.86 (0.27-2.75) |  | 0.95 (0.55-1.63) |  |
|  | Cocaine use, ever |  |  | 1.97 (1.28-3.02) | 0.002 |
|  | Cardiovascular disease | 2.03 (1.09-3.80) | 0.03 | 1.72 (1.03-2.86) | 0.04 |
|  | Bacterial pneumonia, ever | 1.87 (1.04-3.36) | 0.04 | 1.85 (1.35-2.53) | 0.0001 |
|  | *Pneumocystis*, ever |  |  | 1.65 (1.16-2.36) | 0.01 |
|  |  |  |  |  |  |
| Sleep apnea | HAART use (ref=Never) |  | 0.26 |  | 0.26 |
|  | Past | 2.44 (0.65-9.22) |  | 2.32 (0.58-9.26) |  |
|  | Current | 1.49 (0.41-5.36) |  | 2.92 (0.80-10.6) |  |
|  | BMI (per 3 units) | 1.42 (1.26-1.59) | <0.001 | 1.22 (1.13-1.32) | <0.001 |
|  | Age (per 5 Years) | 1.30 (1.13-1.50) | <0.001 |  |  |
|  |  |  |  |  |  |
| Pulmonary hypertension **^2^** | HAART use (ref=Never/Past) |  | 0.71 |  | 0.28 |
|  | Current | 1.87 (0.07-49.7) |  | 2.48 (0.47-13.1) |  |
|  | Viral Load | 1.55 (1.00-2.40) | 0.05 |  |  |
|  | Nadir CD4 count (per 200 counts) | 3.55 (1.02-12.3) | 0.05 |  |  |
|  | Cardiovascular disease |  |  | 5.48 (1.40-21.4) | 0.02 |

Models controlled for age, race, smoking status, pack-years smoked, and intravenous drug use, CVD, pneumonia and history of PCP.

A variable with ‘blank’ indicates not significant.

**^1^** MACS-Oxygen use/Bronchoscopy - HAART use was classified into two groups only: Never/Past vs. Current, because there were zero frequencies in one of the groups.

**^2^** Pulmonary hypertension - HAART and smoking status were classified into two groups only: Never/former vs. Current for MACS and WIHS.

MACS - Multicenter AIDS Cohort Study; WIHS - Women’s Interagency HIV Study; PR - Prevalent ratio; CI - confidence intervals; HAART – highly active antiretroviral therapy; BMI – body mass index; ICU – injection drug use; COPD - chronic obstructive pulmonary disease.

Additional file 1: Table S4a. Proportion of patients with prevalent symptoms, diagnostic testing, and diagnoses comparing HIV-infected and HIV-uninfected by smoking status - **MACS**

|  | **Never Smokers (n=480)** | | | **Former Smokers (n=894)** | | | **Current Smokers (n=501)** | | |
| --- | --- | --- | --- | --- | --- | --- | --- | --- | --- |
|  | **HIV -** | **HIV +** | **p-value** | **HIV -** | **HIV +** | **p-value** | **HIV -** | **HIV +** | **p-value** |
| **Symptoms** |  |  |  |  |  |  |  |  |  |
| Cough, n (%) | 74 (29.2) | 73 (33.3) | 0.33 | 159 (32.7) | 131 (34.1) | 0.66 | 137 (63.7) | 170 (63.2) | 0.90 |
| Dyspnea, n (%) | 14 (5.5) | 22 (10.1) | 0.05 | 48 (9.7) | 52 (13.3) | 0.09 | 31 (13.9) | 49 (18.1) | 0.20 |
| Wheezing, n (%) | 19 (7.4) | 20 (9.1) | 0.49 | 52 (10.5) | 50 (12.8) | 0.28 | 55 (25.0) | 67 (24.4) | 0.87 |
| Phlegm production, n (%) | 67 (26.2) | 63 (28.8) | 0.53 | 120 (24.4) | 119 (30.3) | 0.04 | 102 (46.8) | 128 (46.2) | 0.89 |
| Any symptom, n (%) | 105 (41.2) | 102 (46.8) | 0.22 | 217 (44.6) | 192 (50.0) | 0.11 | 154 (70.6) | 199 (73.2) | 0.54 |
| Inhaler use, n (%) | 46 (17.8) | 50 (22.7) | 0.18 | 108 (21.8) | 115 (29.2) | 0.01 | 60 (27.0) | 81 (29.4) | 0.55 |
| Oxygen use, n (%) | 3 (1.2) | 6 (1.8) | 0.55 | 4 (0.8) | 7 (1.8) | 0.19 | 3 (1.4) | 5 (1.8) | 0.69 |
| **Diagnostic testing** |  |  |  |  |  |  |  |  |  |
| Chest computed tomography, n (%) | 54 (21.6) | 56 (26.8) | 0.19 | 103 (21.9) | 127 (34.1) | <0.001 | 67 (31.0) | 107 (40.7) | 0.03 |
| Bronchoscopy, n (%) | 4 (1.2) | 14 (6.4) | 0.01 | 17 (3.4) | 29 (7.4) | 0.01 | 6 (2.8) | 14 (5.2) | 0.18 |
| Pulmonary function testing, n (%) | 92 (37.3) | 79 (37.2) | 0.99 | 192 (40.6) | 158 (40.8) | 0.94 | 81 (38.0) | 101 (37.1) | 0.84 |
| Sputum sample, n (%) | 10 (3.9) | 26 (12.0) | 0.001 | 33 (6.9) | 75 (19.4) | <0.001 | 8 (3.6) | 52 (19.0) | <0.001 |
| Polysomnography, n (%) | 39 (15.2) | 41 (18.5) | 0.34 | 86 (17.3) | 70 (17.7) | 0.88 | 26 (11.8) | 43 (15.6) | 0.22 |
| Echocardiogram, n (%) | 72 (29.4) | 60 (28.0) | 0.75 | 158 (32.9) | 124 (32.3) | 0.84 | 40 (18.7) | 72 (26.5) | 0.04 |
| **Diagnoses** |  |  |  |  |  |  |  |  |  |
| Asthma, n (%) | 28 (10.9) | 27 (12.2) | 0.65 | 60 (12.1) | 60 (15.3) | 0.16 | 30 (13.5) | 35 (12.7) | 0.78 |
| COPD, n (%) | 8 (3.1) | 13 (5.9) | 0.14 | 32 (6.5) | 29 (7.4) | 0.59 | 24 (10.8) | 30 (10.9) | 0.95 |
| Chronic bronchitis, n (%) | 7 (2.7) | 12 (5.4) | 0.13 | 27 (5.5) | 22 (5.6) | 0.94 | 19 (8.5) | 22 (8.1) | 0.85 |
| Sleep apnea, n (%) | 25 (9.7) | 28 (12.8) | 0.28 | 64 (12.9) | 45 (11.5) | 0.52 | 17 (7.6) | 20 (7.3) | 0.88 |
| Pulmonary hypertension, n (%) | -- | -- | -- | 2 (0.4) | 2 (0.5) | 0.81 | 3 (1.4) | 2 (0.7) | 0.49 |
| Interstitial pulmonary fibrosis, n (%) | -- | 1 (0.5) | -- | -- | 1 (0.3) | -- | -- | -- | -- |
| Sarcoidosis, n (%) | 2 (0.8) | -- | -- | -- | 2 (0.5) | -- | 2 (0.9) | -- | -- |
| Pulmonary embolism, n (%) | 3 (1.2) | 4 (1.8) | 0.56 | -- | 3 (0.8) | -- | 2 (0.9) | 2 (0.7) | 0.83 |

COPD - chronic obstructive pulmonary disease; HIV+ - HIV-infected; HIV- - HIV-uninfected.

Additional file 1: Table S4b. Proportion of patients with prevalent symptoms, diagnostic testing, and diagnoses comparing HIV-infected and HIV-uninfected by smoking status - **WIHS**

|  | **Never Smokers (n=577)** | | | **Former Smokers (n=543)** | | | **Current Smokers (n=835)** | | |
| --- | --- | --- | --- | --- | --- | --- | --- | --- | --- |
|  | **HIV -** | **HIV +** | **p-value** | **HIV -** | **HIV +** | **p-value** | **HIV -** | **HIV +** | **p-value** |
| **Symptoms** |  |  |  |  |  |  |  |  |  |
| Cough, n (%) | 57 (42.2) | 178 (40.4) | 0.71 | 75 (54.0) | 200 (50.0) | 0.41 | 200 (67.8) | 372 (69.0) | 0.72 |
| Dyspnea, n (%) | 28 (20.7) | 90 (20.5) | 0.94 | 41 (29.5) | 147 (36.6) | 0.13 | 140 (47.5) | 266 (49.4) | 0.60 |
| Wheezing, n (%) | 12 (8.9) | 55 (12.5) | 0.25 | 22 (15.8) | 80 (19.9) | 0.29 | 93 (31.5) | 205 (38.1) | 0.05 |
| Phlegm production, n (%) | 23 (17.0) | 108 (24.6) | 0.06 | 52 (37.4) | 158 (39.3) | 0.69 | 147 (50.0) | 300 (55.7) | 0.12 |
| Any symptom, n (%) | 71 (52.6) | 222 (50.5) | 0.66 | 89 (64.0) | 259 (64.6) | 0.90 | 225 (76.5) | 437 (81.2) | 0.11 |
| Inhaler use, n (%) | 33 (24.4) | 99 (22.5) | 0.63 | 46 (33.1) | 157 (39.1) | 0.21 | 133 (45.1) | 297 (55.1) | 0.01 |
| Oxygen use, n (%) | 1 (0.7) | 7 (1.6) | 0.46 | -- | 10 (2.5) | -- | 5 (1.7) | 17 (3.2) | 0.21 |
| **Diagnostic testing** |  |  |  |  |  |  |  |  |  |
| Chest computed tomography, n (%) | 8 (5.9) | 42 (9.6) | 0.19 | 16 (11.5) | 80 (19.9) | 0.02 | 47 (15.9) | 98 (18.3) | 0.40 |
| Bronchoscopy, n (%) | 4 (3.0) | 15 (3.4) | 0.80 | 3 (2.6) | 15 (3.7) | 0.37 | 7 (2.4) | 25 (4.6) | 0.10 |
| Pulmonary function testing, n (%) | 22 (16.3) | 82 (18.6) | 0.54 | 37 (26.6) | 110 (27.4) | 0.86 | 92 (31.3) | 173 (32.1) | 0.81 |
| Sputum sample, n (%) | 4 (3.0) | 36 (8.2) | 0.03 | 4 (2.9) | 81 (20.2) | <0.001 | 31 (10.5) | 160 (29.7) | <0.001 |
| Polysomnography, n (%) | 10 (7.4) | 24 (5.5) | 0.40 | 10 (7.2) | 33 (8.2) | 0.70 | 17 (5.8) | 25 (4.7) | 0.48 |
| Echocardiogram, n (%) | 14 (10.4) | 56 (12.7) | 0.46 | 24 (17.3) | 88 (21.9) | 0.25 | 49 (16.6) | 75 (14.0) | 0.31 |
| **Diagnoses** |  |  |  |  |  |  |  |  |  |
| Asthma, n (%) | 16 (11.8) | 51 (11.6) | 0.93 | 22 (15.8) | 86 (21.4) | 0.16 | 108 (36.9) | 182 (33.8) | 0.37 |
| COPD, n (%) | 5 (3.7) | 31 (7.1) | 0.16 | 13 (9.4) | 52 (13.0) | 0.26 | 41 (14.0) | 129 (23.9) | 0.001 |
| Chronic bronchitis, n (%) | 5 (3.7) | 28 (6.4) | 0.24 | 13 (9.4) | 42 (10.5) | 0.71 | 36 (12.2) | 110 (20.4) | 0.003 |
| Sleep apnea, n (%) | 3 (2.2) | 11 (2.5) | 0.85 | 3 (2.2) | 24 (6.0) | 0.07 | 10 (3.4) | 25 (4.6) | 0.40 |
| Pulmonary hypertension, n (%) | 1 (0.7) | 4 (0.9) | 0.85 | -- | 5 (1.2) | -- | 3 (1.0) | 5 (0.9) | 0.89 |
| Interstitial pulmonary fibrosis, n (%) | -- | 2 (0.5) | -- | -- | 1 (0.3) | -- | -- | -- | -- |
| Sarcoidosis, n (%) | -- | 3 (0.7) | -- | -- | 7 (1.7) | -- | 1 (0.3) | 3 (0.6) | 0.66 |
| Pulmonary embolism, n (%) | -- | 1 (0.2) | -- | 1 (0.7) | 3 (0.8) | 0.97 | 1 (0.3) | 6 (1.1) | 0.24 |

COPD - chronic obstructive pulmonary disease; HIV+ - HIV-infected; HIV- - HIV-uninfected.

Additional file 1: Table S5a: Final adjusted Poisson regression models of baseline factors associated with prevalent pulmonary outcomes in the MACS cohort.

| Significant factors | Adjusted PR | 95% CI | p-value |
| --- | --- | --- | --- |
| **Asthma** |  |  |  |
| HIV status | 1.08 | 0.83-1.40 | 0.57 |
| Age (units=5) | 0.89 | 0.83-0.95 | 0.001 |
| **Sleep apnea** |  |  |  |
| HIV status | 1.50 | 1.11-2.03 | 0.01 |
| BMI (units=3) | 1.38 | 1.30-1.46 | <.0001 |
| Age (units=5) | 1.24 | 1.14-1.34 | <.0001 |
| **Any COPD** |  |  |  |
| HIV status | 1.22 | 0.86-1.73 | 0.26 |
| Pack years (units=10) | 1.17 | 1.08-1.27 | <.0001 |
| **Cough** |  |  |  |
| HIV status | 1.10 | 0.94-1.28 | 0.23 |
| Smoking status |  |  | <.0001 |
| Current | 1.88 | 1.47-2.40 |  |
| Former | 1.02 | 0.83-1.25 |  |
| IDU ever | 0.80 | 0.64-1.00 | 0.05 |
| Pack Years (units=10) | 1.04 | 1.00-1.08 | 0.04 |
| BMI (units=3) | 1.12 | 1.04-1.20 | 0.001 |
| **Dyspnea** |  |  |  |
| HIV status | 1.33 | 1.01-1.75 | 0.04 |
| Hispanic | 1.75 | 1.15-2.65 | 0.01 |
| Pack years (units=10) | 1.08 | 1.00-1.16 | 0.04 |
| **Wheezing** |  |  |  |
| HIV status | 1.13 | 0.88-1.46 | 0.32 |
| Smoking status |  |  | 0.0032 |
| Current | 1.90 | 1.23-2.96 |  |
| Former | 1.19 | 0.81-1.74 |  |
| Pack years (units=10) | 1.13 | 1.06-1.21 | 0.0002 |
| BMI (units=3) | 1.16 | 1.08-1.24 | <.0001 |
| Age (units=5) | 0.91 | 0.85-0.98 | 0.01 |
| **Sputum** |  |  |  |
| HIV status | 1.13 | 0.96-1.33 | 0.15 |
| Smoking status |  |  | <.0001 |
| Current | 1.43 | 1.09-1.88 |  |
| Former | 0.89 | 0.71-1.11 |  |
| Cocaine use ever | 1.21 | 1.02-1.44 | 0.03 |
| Pack years (units=10) | 1.06 | 1.02-1.11 | 0.01 |
| **Any symptom** |  |  |  |
| HIV status | 1.15 | 1.01-1.31 | 0.04 |
| Smoking status |  |  | <.0001 |
| Current | 1.53 | 1.23-1.89 |  |
| Former | 1.03 | 0.87-1.23 |  |
| Pack years (units=10) | 1.04 | 1.01-1.08 | 0.02 |
| BMI (units=3) | 1.06 | 1.02-1.10 | 0.002 |
| **Pulmonary function tests** |  |  |  |
| HIV status | 1.06 | 0.91-1.24 | 0.44 |
| Hispanic | 0.64 | 0.46-0.91 | 0.01 |
| Age (units=5) | 1.05 | 1.01-1.10 | 0.02 |

* Adjusted variables are HIV Status, black, smoking status, IV drug use ever, Pack Years and age.

BMI – body mass index; IDU – injection drug use

Additional file 1: Table S5b: Final adjusted Poisson regression models of factors associated with prevalent pulmonary outcomes in the WIHS cohort.

| Significant factors | Adjusted PR | 95% CI | p-value |
| --- | --- | --- | --- |
| **Asthma** |  |  |  |
| HIV status | 1.05 | 0.86-1.28 | 0.65 |
| Smoking status (ref=Never) |  |  | <.0001 |
| Current | 2.03 | 1.37-3.00 |  |
| Former | 1.23 | 0.86-1.75 |  |
| Cocaine use ever | 1.65 | 1.27-2.14 | 0.0001 |
| BMI (units=3) | 1.07 | 1.03-1.10 | 0.0002 |
| **Sleep apnea** |  |  |  |
| HIV status | 1.88 | 1.07-3.31 | 0.03 |
| Black | 0.52 | 0.30-0.88 | 0.01 |
| Hispanic | 0.34 | 0.16-0.69 | 0.003 |
| BMI (units=3) | 1.25 | 1.18-1.34 | <.0001 |
| **Any COPD** |  |  |  |
| HIV status | 1.56 | 1.16-2.09 | 0.003 |
| Black | 0.65 | 0.48-0.87 | 0.004 |
| Hispanic | 0.70 | 0.49-0.99 | 0.04 |
| Cocaine use ever | 1.90 | 1.31-2.75 | 0.001 |
| Pack years (units=10) | 1.24 | 1.06-1.45 | 0.01 |
| BMI (units=3) | 1.07 | 1.02-1.12 | 0.01 |
| Age (units=5) | 1.10 | 1.01-1.20 | 0.03 |
| **Cough** |  |  |  |
| HIV status | 0.99 | 0.87-1.13 | 0.87 |
| Smoking status (ref=Never) |  |  | 0.01 |
| Current | 1.37 | 1.08-1.75 |  |
| Former | 1.07 | 0.87-1.32 |  |
| BMI (units=3) | 1.03 | 1.01-1.05 | 0.01 |
| **Dyspnea** |  |  |  |
| HIV status | 1.07 | 0.91-1.27 | 0.40 |
| Smoking status (ref=Never) |  |  | 0.01 |
| Current | 1.57 | 1.15-2.15 |  |
| Former | 1.23 | 0.94-1.62 |  |
| Cocaine use ever | 1.35 | 1.09-1.67 | 0.01 |
| BMI (units=3) | 1.06 | 1.03-1.09 | 0.0001 |
| **Wheezing** |  |  |  |
| HIV status | 1.23 | 1.02-1.56 | 0.03 |
| Smoking status (ref=Never) |  |  | <.0001 |
| Current | 1.82 | 1.22-2.71 |  |
| Former | 1.04 | 0.73-1.50 |  |
| Cocaine use ever | 1.64 | 1.25-2.13 | 0.0003 |
| BMI (units=3) | 1.08 | 1.04-1.11 | <.0001 |
| **Sputum** |  |  |  |
| HIV status | 1.11 | 0.95-1.31 | 0.19 |
| Smoking status (ref=Never) |  |  | <.0001 |
| Current | 1.89 | 1.41-2.54 |  |
| Former | 1.41 | 1.09-1.82 |  |
| **Any symptom** |  |  |  |
| HIV status | 1.03 | 0.91-1.17 | 0.59 |
| Smoking status (ref=Never) |  |  | 0.01 |
| Current | 1.40 | 1.12-1.74 |  |
| Former | 1.14 | 0.95-1.37 |  |
| BMI (units=3) | 1.03 | 1.01-1.05 | 0.003 |
| **Pulmonary function tests** |  |  |  |
| HIV status | 1.01 | 0.83-1.22 | 0.96 |
| Hispanic | 0.76 | 0.58-0.98 | 0.03 |
| Cocaine use ever | 1.44 | 1.13-1.84 | 0.003 |
| BMI (units=3) | 1.04 | 1.00-1.07 | 0.03 |
| Age (units=5) | 1.08 | 1.02-1.14 | 0.01 |

* Adjusted variables are HIV Status, black, smoking status, IV drug use ever, Pack Years and age.

BMI – body mass index; IDU – injection drug use
